# Geometrical assembly of ultrastable protein templates for nanomaterials

**DOI:** 10.1038/ncomms11771

**Published:** 2016-06-01

**Authors:** Dominic J. Glover, Lars Giger, Steve S. Kim, Rajesh R. Naik, Douglas S. Clark

**Affiliations:** 1 Department of Chemical and Biomolecular Engineering, University of California, Berkeley, California 94720, USA; 2 Materials and Manufacturing Directorate, Air Force Research Laboratory, Wright-Patterson Air Force Base, Dayton, Ohio 45433, USA

## Abstract

The fabrication of nanoscale devices requires architectural templates on which to position functional molecules in complex arrangements. Protein scaffolds are particularly promising templates for nanomaterials due to inherent molecular recognition and self-assembly capabilities combined with genetically encoded functionalities. However, difficulties in engineering protein quaternary structure into stable and well-ordered shapes have hampered progress. Here we report the development of an ultrastable biomolecular construction kit for the assembly of filamentous proteins into geometrically defined templates of controllable size and symmetry. The strategy combines redesign of protein–protein interaction specificity with the creation of tunable connector proteins that govern the assembly and projection angles of the filaments. The functionality of these nanoarchitectures is illustrated by incorporation of nanoparticles at specific locations and orientations to create hybrid materials such as conductive nanowires. These new structural components facilitate the manufacturing of nanomaterials with diverse shapes and functional properties over a wide range of processing conditions.

## Abstract (web summary)

Protein nanotechnology for the fabrication of protein-based nanoscale devices is gaining momentum but assembling well-defined three-dimensional shapes is still challenging. Here, the authors use an existing prefoldin assembled system to design a template for the construction of geometrically constrained structures.

## Graphical abstract

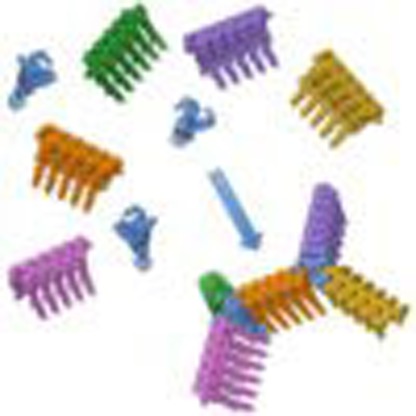

Advances in nanotechnology call for new applications that push the limits of existing fabrication methodology. These applications envision the precise placement of functional features on the nanoscale that can be patterned over large surface areas. Biological-based templates offer many advantages for nanofabrication, such as the ability to self-assemble, defined sequence-structure relationships and self-correcting mechanisms. Although peptides, proteins and DNA have all been used as scaffolds to precisely position molecular components on the nanoscale, engineered protein templates have not yet reached a level of sophistication approaching that of their most advanced DNA counterparts, for example, the exquisite patterns known as DNA origami^1^,^2^. Current protein templates are generally limited to naturally self-assembling systems such as viral coat proteins^3^ owing to difficulties associated with generating new protein shapes. The difficulties range from redesigning protein–protein interfaces for the folding of unique quaternary structures^4^,^5^,^6^, to overcoming environmental limitations such as a narrow range of stability or high energy requirements for assembly.

The filamentous protein γ-prefoldin (γPFD), isolated from the hyperthermophile *Methanocaldococcus jannaschii*, has the potential to create a protein scaffold system that circumvents conventional limitations. A variant of the prefoldin family of chaperones, γPFD assembles into filamentous structures (Fig. 1a) to assist with maintaining the correct conformation of endogenous proteins in the extreme environments inhabited by its deep-sea archaeal host^7^. The γPFD monomers assemble into filaments through association domains composed of β-sheets (designated X1 and X2; Fig. 1b), with the remainder of the protein protruding outwards in a coiled-coil domain to create a brush-like structure (Fig. 1c). Filaments of γPFD vary in length, in some cases exceeding 3 μm, and exhibit remarkable stability with a midpoint of the transition (*T*_M_) of 93 °C (ref. 8). Furthermore, unlike biological filaments such as actin and microtubules, filaments of γPFD have no polarity, so end-to-end joining of a single filament should be possible.

The malleable and distinct modular architecture of the γPFD filament provides an ideal starting point to construct novel protein architectures for myriad applications. Importantly, the interface between each γPFD subunit is pliant and amenable to structural modification^9^; the β-sheet oligomerization domains and the coiled-coils of the subunits are physically separate and can be modified independently for expanded function^10^; and the assembled filament is extremely stable to thermal and solvent-induced denaturation^8^. The inherent molecular chaperone function of the filament^11^ may also serve to stabilize and re-activate enzymes in denaturing conditions such as during production of composite materials and enzyme–nanomaterial conjugates. Furthermore, progress has been made to gain control over γPFD filament elongation through the creation of a capping protein called TERM (thermophilic extension resistant mutant) to control filament length^9^. The TERM contains mutations in the X2 β-sheet region of γPFD (Fig. 1b) that prevent filament elongation but still enable γPFD binding through the functional X1, thereby allowing TERM to attach to the termini of nascent filaments and cap their extension.

While extensive effort has focused on tweaking existing two- or three-dimensional assembled systems, γPFD provides the opportunity to develop structures unconstrained by natural architectures by starting with a one-dimensional (1D) foundation and expanding it in additional dimensions. We therefore proposed to exploit the unique advantages of γPFD by utilizing its structure as the starting point to engineer templates that self-assemble from interconnecting parts into geometrically constrained structural templates. The functionality of the templates was demonstrated by the precise positioning of gold nanoparticles in regular patterns to fabricate highly conductive nanowires with specific lengths and architectures.

## Results

### Design and creation of connector proteins

**Figure 1:**
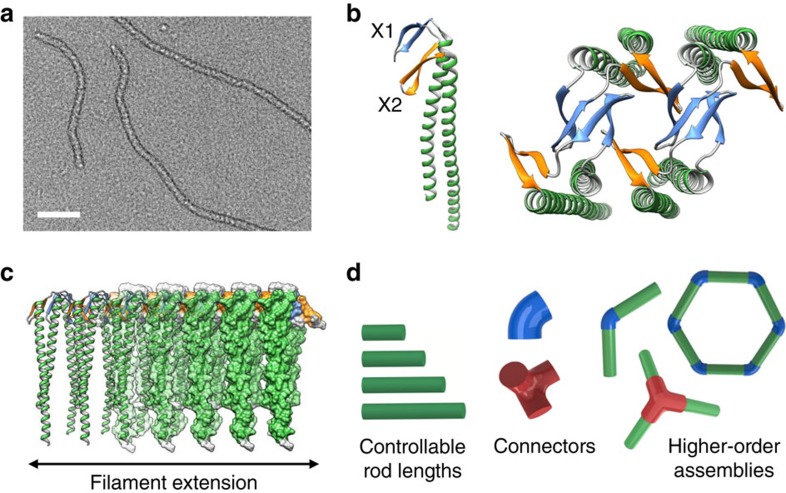
Structure and design principles for the engineering of filaments into higher-order structures. (**a**) TEM image of γPFD filaments (scale bar, 100 nm). (**b**) The γPFD subunit is composed of two β-sheets, designated X1 and X2, which are flanked by helical regions that form a coiled-coil. (**c**) The β-sheet domains are responsible for the assembly of the subunits into long filaments. (**d**) Schematics for the design and assembly of filament rods and connectors into higher-order structures.

**Figure 2:**
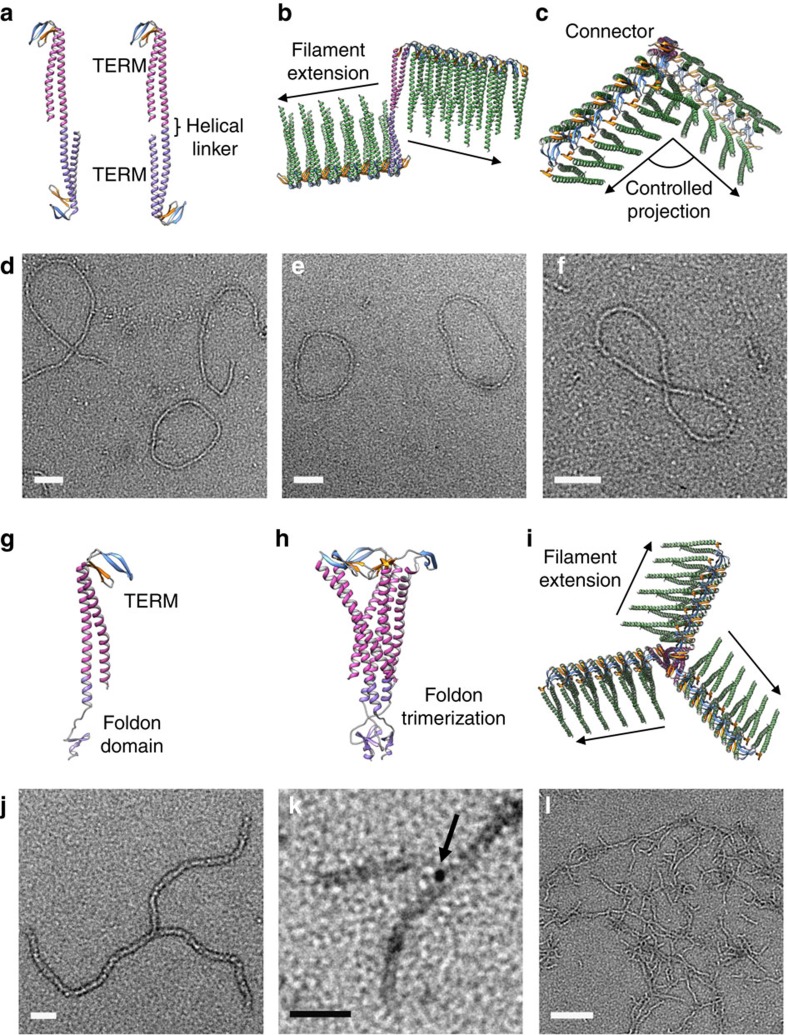
Geometrical assembly of γPFD filaments. (**a**) Two-way connectors were designed by fusion of two TERM monomers. Each connector is designed to impart a specific angle between filaments. (**b**) Filaments of γPFD elongate from the connector in two directions, (**c**) at a specific angle imparted by the orientation of the TERM X1 β-sheet interfaces of the connector. (**d**) TEM image of two-way connectors assembled with γPFD to create self-closing structures either (**e**) ovoidal or (**f**) figure-eight in shape (scale bars, 100 nm). (**g**) A three-way connector was created by fusion of TERM with the foldon trimerization domain. (**h**) A trimer of TERM-foldon that (**i**) assembles with γPFD to elongate outwards from the TERM β-sheet interfaces to create a pinwheel structure. (**j**) TEM of the three-way connector assembled with three γPFD filaments into a pinwheel shape with arms of uniform length and (**k**) its structure confirmed by the positioning of a gold nanoparticle (arrow) covalently attached to a C-terminal cysteine on the connector (scale bars, 50 nm). (**l**) Cross-linking of filaments using a high stoichiometric ratio of three-way connector to γPFD (scale bar, 200 nm).

The initial aim was to develop a standardized biomolecular construction set for joining individual filaments together using angled connector proteins into geometrically constrained structures (Fig. 1d). Our approach exploits the intrinsic ability of the monomeric building blocks to self-assemble, and extends their natural capacity through rational design of subunit interactions. Two-way connector proteins were created by genetic fusion of two complementary TERM monomers using a helical linker between the individual coiled-coil domains (Fig. 2a). The use of TERM ensures that γPFD filament growth only occurs in a single direction from the functional X1 site of each TERM within the fusion protein (Fig. 2b). The helical linker joining each TERM subunit has a predictable turn of 100° per amino acid residue. Thus, a unique advantage of this system is that addition or subtraction of helical length between the TERM domains will change the projection angle of the adjoining filaments. Semi-rigid linker sequences have been used previously to join oligomerization domains together for controlled assembly of symmetrical nanostructures^12^,^13^, and in our approach will serve to modulate the geometry of the final structures. An initial two-way connector was created that was designed to impart an angle of 101° between the two attached filaments (Fig. 2c).

A three-way connector was created using a trimerization domain called foldon in fusion with TERM as a method to join together three individual γPFD filaments. The foldon domain is a small 27-residue trimeric structure found at the C-terminus of the helical bacteriophage T4 fibritin protein. The foldon domain is compatible with our strategy due to its high thermal stability^14^, and the helical structure of the N-terminal region of foldon enables it to be directly joined to the C-terminal coiled-coil of γPFD as a continuous helix (Fig. 2g). The TERM-foldon protein is designed to trimerize into a three-way connector (Fig. 2h) that is capable of attaching to three individual filaments that extend outwards to create a pinwheel-like structure (Fig. 2i).

The γPFD and connector proteins were expressed in *Escherichia coli* and purified by ion-exchange or affinity chromatography (Supplementary Fig. 1). Far-ultraviolet circular dichroism scans of γPFD and connector proteins at room temperature had near-identical helical conformations, as would be expected for correctly folded connector proteins that are structurally similar to γPFD (Supplementary Fig. 2). The γPFD and two-way connectors retained their secondary structure during a thermal ramp to 100 °C, while the three-way connector unfolded with a *T*_M_ at 74.1±0.2 °C.

To examine the trimerization of the three-way connector and the thermal stability of the resulting complex, we took advantage of the foldon domain's resistance to SDS-induced denaturation^14^. During SDS–PAGE, the TERM-foldon migrated as a trimer when it was not heated before electrophoretic separation (Supplementary Fig. 3). Incubation of the TERM-foldon at increasing temperatures before electrophoresis resulted in denaturation of the trimer into monomeric subunits. Densitometric analysis of the SDS–PAGE revealed a *T*_M_ of 63.3±0.5 °C for the dissociation of the trimer into monomers in the presence of 2% SDS. To examine the thermal stability of the TERM-foldon trimer without the presence of a denaturant, the fluorescence emission maxima of the sole tryptophan in the connector was measured as a function of increasing temperature. The tryptophan is located in the first β-sheet of the foldon domain (Fig. 2g) and had a maximum fluorescence emission of 328 nm at 25 °C (Supplementary Fig. 3), as expected from a tryptophan residue that is buried within the trimeric interface. During a thermal ramp, the fluorescence emission maximum was redshifted to 356 nm, suggesting the local environment of the tryptophan became solvent exposed. A curve fit of this unfolding process revealed the TERM-foldon trimer to have a *T*_M_ of 84.1±0.2 °C, which is consistent with previous measurements of foldon^15^. The three-way connector appears to undergo a two-stage thermal unfolding with the helical domains dissociating before the foldon trimizeration domain. We have shown previously that the thermal stability of γPFD can be strengthened by increasing the coiled-coil hydrophobicity^8^. The same strategy could also be applied to potentially increase the thermal stability of the TERM-foldon connector. Regardless, the high thermal stability exhibited by both the two- and three-way connectors in conjunction with the γPFD should enable their use in biomaterial applications that require exceptional stability and robustness.

### Assembly of connectors and filaments into defined shapes

The connectors were assembled with γPFD in a controlled manner by first denaturing the proteins in 8 M guanidinium-HCl and combining the proteins in various molar ratios, followed by rapid dilution and refolding in an aqueous buffer. The propensity of the connectors to attach to γPFD subunits and extend filaments was confirmed using a protein–protein interaction pull-down assay^11^ (Supplementary Fig. 4). The connector–filament assemblies were imaged by negative-stain transmission electron microscopy (TEM). At a molar ratio 1:400 of two-way connector to γPFD subunits we observed self-closing loop topology (Fig. 2d). The loops had an average circumference of 803±234 nm, and an average diameter of 232±75 nm (Fig. 2e). With a spacing of 2.3 nm per γPFD dimer along the longitudinal axis (Fig. 1c), each loop contains an average of 698 subunits. A number of figure-eight structures were also observed (Fig. 2f). The length of these assemblies was significantly longer than the loop assemblies, at 1,320±165 nm, which corresponds to 1,148 subunits. Filaments of γPFD have a long persistence length (a measure of filament stiffness) of 730 nm (ref. 10), and filaments almost never naturally self-close. Incorporation of a two-way connector into filaments presumably lowers the free energy change for closure and overcomes the strain energy required to generate bends in the filament.

Assembly of the three-way connectors with γPFD resulted in the creation of pinwheel structures matching the design model, with three filaments linked through a central hub (Fig. 2j). At a molar ratio of 1:100 of connector to γPFD, the average length of the attached filaments was 122±54 nm, which corresponds to an average of 106 subunits per filament. Lowering the concentration of connector to γPFD (1:300) resulted in longer filaments attached to the connector, suggesting that average filament length can be controlled by titrating connector to γPFD. To confirm that the connector was at the centre of the pinwheel structures, a TERM-foldon was created that contained a C-terminal cysteine that was selectively labelled with a gold particle via maleimide chemistry. The nanoparticle was shown to be located at only the centre of the pinwheel assemblies, thereby confirming that the structures are not two separate perpendicular filaments (Fig. 2k). This labelling strategy also demonstrates that functional molecules may be attached to specific locations on the connector–filament assemblies. Refolding γPFD with higher concentration of the three-way connector (1:50) resulted in extensive cross-linking between filaments (Fig. 2l), presumably due to filaments linking between multiple connectors. It was apparent that greater control over assembly may be required to build more complex and higher-order shapes.

The coiled-coil regions of prefoldins, including the γPFD, have been shown to be structurally flexible compared with the immobile β-sheet barrel backbone^11^,^16^. Within the connector proteins, the coiled-coils serve to bridge the protein–protein interaction domains (Fig. 2a,g); therefore, this flexibility may affect the filament projection angle and assembly of the filament. To provide atomistic insights into the structure and thermal-induced flexibility of the connector proteins, molecular dynamic simulations were performed. With the exception of the short hydrogen bonded turns that link the β-sheets together, the residues of the two-way connector were moderately flexible during the 300 K simulation (Supplementary Fig. 5). During the 353 K simulation, however, there was an increase in the flexibility of the connector, and in particular, the α-helical region that joins the two TERM subunits. At elevated temperatures, this region appeared to function as a molecular ‘hinge' that adds structural plasticity to the connector. This suggests that temperature could be used to control the flexibility and filament projection angle of the connector, an approach that is compatible with the high thermal stability of the connector and γPFD filaments.

The three-way connector also displayed increased structural flexibility during simulation at 353 K compared with 300 K; however, this occurred predominately in the hydrogen bonded turns in both the TERM and the foldon domain. Unlike the two-way connector, the coiled-coils remained relatively inflexible, due to the coiled-coils being bundled together and spatially constrained. This structural rigidity also meant that elevated temperature did not significantly alter the projection angle of the X1 β-sheets. In addition, the inactivated X2 β-sheets stayed in close confinement, while the β-turns jostled with each other (Supplementary Fig. 5). Molecular modelling of this region suggested that increasing the size of the X2 β-sheet would force apart the coiled-coils at the TERM interfaces. The resulting bend in the coiled-coils would alter the Z-projection angle of the X1 interface, effectively extending filaments at a downwards angle. Engineering the extension angle of the three-way connectors may provide a method to control the assembly of connectors and filaments in higher-order structures, for example, a cubic assembly.

### Protein calligraphy by controlled connector assembly

**Figure 3:**
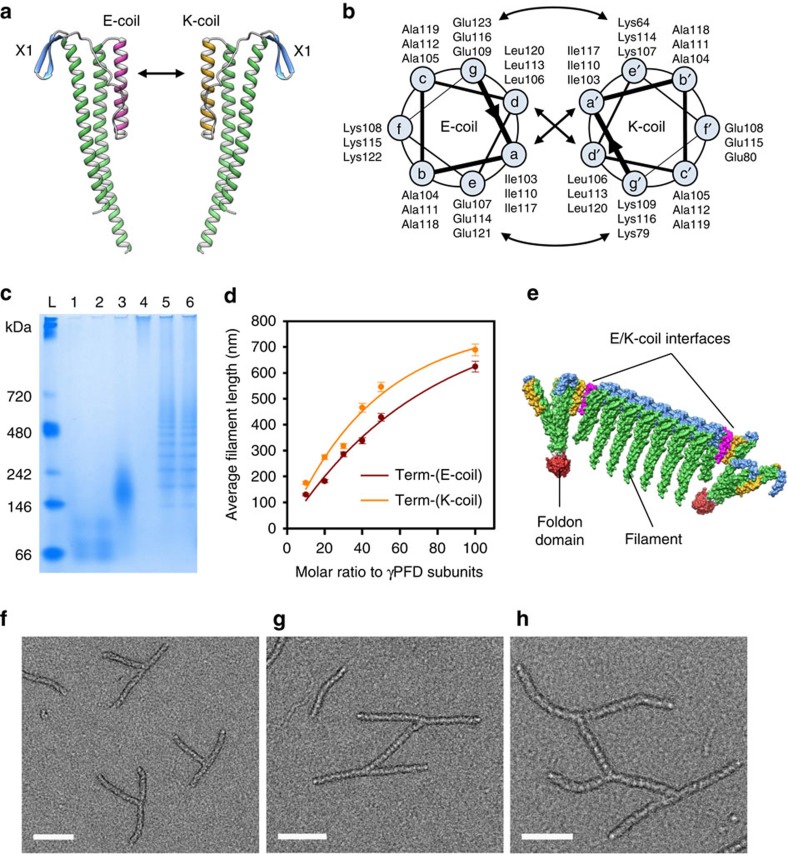
Engineering of the γPFD interface for controlled connector assembly. (**a**) *De novo* protein models of the TERM-(E-coil) and TERM-(K-coil) that are designed to associate together through an engineered heterodimer coiled-coil. (**b**) Helical wheel representation of the E3/K3 heterodimer, in which the coiled-coil is viewed as a cross-section (three letter amino acid code). The interhelical hydrophobic interactions (a–a′, d–d′) and electrostatic interactions (g–e′, e–g′) are denoted with arrows. (**c**) Native gel electrophoresis showing TERM-(E-coil) (lane 1) and TERM-(K-coil) (lane 2) individually or mixed together (lane 3). Filaments of γPFD are too large to run in the gel (lane 4); however, when mixed with TERM-(E-coil) (lane 5) and TERM-(K-coil) (lane 6), the filaments are capped and elongation is inhibited, which produces filaments that vary in the number of subunits. L, protein standard. (**d**) The length of γPFD filaments capped by various ratios of either TERM-(E-coil) or TERM-(K-coil). Results represent the mean±s.e. (*n*=500 filaments). (**e**) A TERM-(E-coil) capped filament attached at the termini of two TERM-(K-coil)-foldon three-way connectors through heterodimeric coiled-coils. (**f**) TEM image of the TERM-(K-coil)-foldon three-way connector assembled with γPFD filaments that are capped with TERM-(E-coil). (**g**–**h**) Increased concentration of the TERM-(E-coil)-foldon results in greater cross-linking between capped filaments in a controlled manner. Scale bars, 100 nm.

Filaments of γPFD are highly variable in length^8^; therefore, it was not surprising that filaments attached to the connectors, for example, the arms of the pinwheel, were non-uniform in length. Furthermore, as the pinwheels are capable of binding to each other, the pinwheels can assemble together into random and undefined assemblages (Fig. 2l). It was evident that greater control over assembly of the connectors and γPFD filaments would be required for the creation of complex and higher-order structures. Previously, we have shown that capped filaments with a narrow distribution of lengths can be created by titrating TERM with γPFD^11^. However, these TERM-capped filaments have non-functional terminal X2 β-sheets and are unable to attach to other filaments or the engineered connectors. We therefore sought to redesign the X2 interface of γPFD to generate ‘bait-prey' pairs that are specific for their binding partner, while still capable of associating with γPFD through the X1 domain to control filament extension. To achieve this aim, we replaced the X2 β-sheet with opposing helical domains that associate together to form a tight heterodimeric coiled-coil (Fig. 3a).

Coiled-coils are attractive building blocks as the rules governing coiled-coil assembly are well understood^17^, which has facilitated the creation of complex protein nanostructures^6^. We chose to use the coiled-coil IAAL E3/K3, which is a *de novo* engineered heterodimer coiled-coil comprising three heptad repeats per subunit that associate together with high affinity and stability^18^,^19^. The IAAL E3/K3 coiled-coil consists of two oppositely charged helical domains (E-coil and K-coil), which contain glutamic acid and lysine residues at positions ‘e' and ‘g' of the heptad repeat, respectively (Fig. 3b). The presence of the oppositely charged glutamic acid and lysine results in strong and specific interhelical electrostatic interactions, which also prevent the E-coil and K-coil from homodimerization. The E-coil and K-coil sequences were substituted into the X2 domain of TERM to create the bait-prey proteins, TERM-(E-coil) and TERM-(K-coil) (Fig. 3a).

Secondary structure comparison of the TERM-(E-coil) and TERM-(K-coil) by circular dichroism showed that the proteins were structurally similar to γPFD and predominately helical with minima near 208 and 222 nm (Supplementary Fig. 6). Combining an equimolar ratio of the TERM-(E-coil) and TERM-(K-coil) resulted in an increase in the ellipticity ratio of [Θ]222 over [Θ]208, which is consistent with an increase in coiled-coil structure that would occur when the E-coil and K-coil helical domains associate together. The TERM-(E-coil) and TERM-(K-coil) were also shown to have the same thermal stability as the γPFD and retained their secondary structure during a thermal ramp to 100 °C (Supplementary Fig. 6).

The specificity of the bait-prey proteins to assemble with each other or γPFD was examined by native PAGE. When refolded together, the TERM-(E-coil) and TERM-(K-coil) formed a complex that was greater in size than the individual proteins (Fig. 3c), suggesting that the proteins specifically bind their partner but not themselves. The filamentous γPFD was too large to separate in the acrylamide gel. However, when γPFD was refolded in the presence of an equimolar amount of either the TERM-(E-coil) or TERM-(K-coil), a laddering effect was observed (Fig. 3c). This is due to the bait-prey proteins becoming incorporated into nascent γPFD filaments, thereby preventing further elongation and producing a distribution of filament lengths.

The affinity of the interaction between the TERM-(E-coil) and TERM-(K-coil) was quantified using a FRET-based assay^19^,^20^, and when fitted with a *B*_max_ of 1 μM (the maximum of bound protein) gave a *K*_d_ of 0.585 μM (Supplementary Fig. 7). This is comparable to the *K*_d_ of 0.457 μM for E3/K3 heterodimer peptides reported previously^19^. The *K*_d_ for the dimerization of TERM subunits through their X1 β-sheet regions was also examined using the FRET assay, which gave a *K*_d_ of 0.622 μM. This *K*_d_ for the X1 dimerization is representative for the interaction affinity of the connectors to γPFD subunits, which also assemble through the X1 β-sheet domains (Fig. 2b,i).

To demonstrate that the bait-prey proteins can be used to control the assembly and length of filaments, the TERM-(E-coil) or TERM-(K-coil) were refolded in the presence of γPFD at varying molar ratios. Imaging of the resulting filaments by TEM showed that the addition of either bait-prey protein produced filament lengths in a concentration-dependent manner (Fig. 3d). Furthermore, all mixtures displayed Gaussian distributions of lengths when plotted on a log_10_ length scale (Supplementary Fig. 8), which is indicative of a step-wise polymerization reaction^9^. These results demonstrate that the bait-prey proteins can be used to create filaments of a desired length.

Next we examined whether these bait-prey-capped filaments could be assembled together using the connector proteins into ordered shapes with specific dimensions. A three-way connector was created that contains the TERM-(K-coil) in fusion with the foldon domain (Fig. 3e). As the X1 β-sheet in the TERM-(K-coil)-foldon is no longer required to attach to γPFD, the domain was inactivated in a similar manner as the X2 of TERM^9^. Seven contiguous residues in the X1 β-sheet were identified to be important for assembly because of spatial proximity and burial of solvent-accessible surface area^21^, and mutated to alanines. In the initial three-way connector, the X1 β-sheets are orientated outwards to attach γPFD and the inactive X2 domains face inwards. However, in the TERM-(K-coil)-foldon connector, the inactivated X1 β-sheet was rotated 200° to enable the K-coil to project outwards (in place of the X2 domain) by the removal of residues from the helical region joining the coiled-coil to the foldon domain (Fig. 3e). This TERM-(K-coil)-foldon connector was shown to be capable of attaching to TERM-(E-coil) using a pull-down assay (Supplementary Fig. 9). We have previously shown that size-exclusion chromatography can be used to reduce the length distribution of TERM-capped γPFD filaments to improve length uniformity^11^. This methodology was used to separate filaments capped with a 1:10 molar ratio of TERM-(E-coil) to γPFD (Fig. 3d) to produce filaments that had an average length of 90 nm and a s.d. of 16 nm. Assembly of the capped filaments with the TERM-(K-coil)-foldon connector produced pinwheel structures with relatively uniform arms (Fig. 3f). Due to the excess of capped filaments, single filaments were also observed. Increasing the ratio of capped filaments to connector to 1:3 produced branched structures (Fig. 3g) with multiple filaments bridging between individual connectors (Fig. 3h). These results demonstrate a versatile approach to control the assembly and overall dimensions of protein templates through addition of varying concentrations of the capped filaments and connectors. The interlocking shapes produced conjure the aesthetic of ‘protein calligraphy.'

### Biotemplated nanowires of controllable dimensions

**Figure 4:**
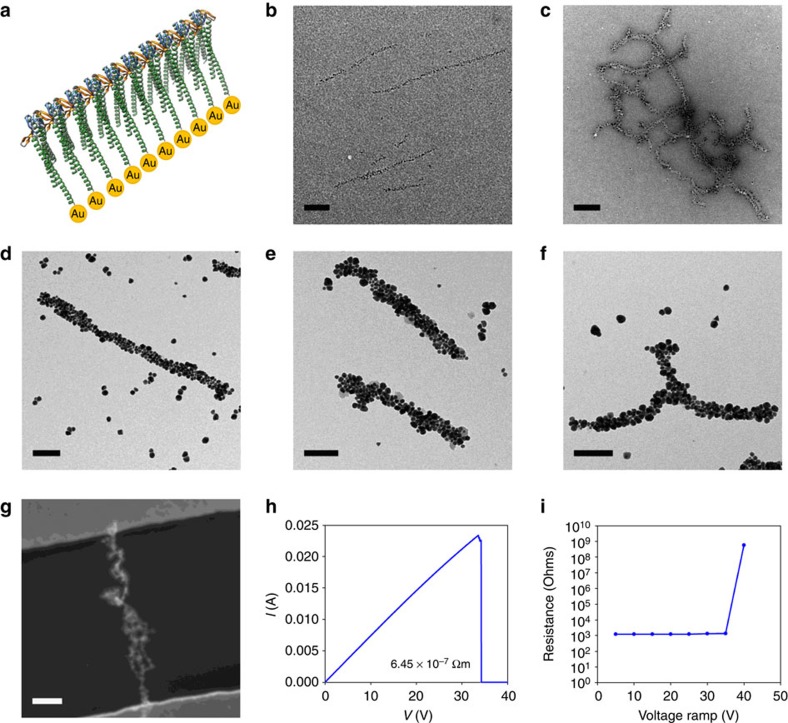
The controlled positioning of nanoparticles on filament templates. (**a**) Schematic diagram of gold nanoparticles attached to the C terminus of γPFD subunits (scale bar, 200 nm). (**b**) TEM image of 1.8-nm and, (**c**), 5-nm Ni-NTA-nanogold arrayed onto filaments of γPFD. (**d**) TEM image of continuous Au nanowires created by deposition of additional gold on filaments with arrayed Ni-NTA nanogold. (**e**) TEM image of TERM-(E-coil) nanowires filaments capped at a 1:50 ratio to produce uniform lengths. (**f**) Conductive nanoarchitecture were created by assembling TERM-(E-coil) capped filaments with a three-way connector and patterned with gold. Scale bars, (**c**–**f**) 100 nm. (**g**) Scanning electron microscopy image of a filament nanowire that bridges the gap between electrodes (scale bar, 500 nm). (**h**) Two-terminal current-voltage *I-V* plot of a γPFD-Au nanowire. (**i**) Resistance versus voltage ramp plot.

Although the fabrication of 1D nanoparticle arrays and electronic components using biological templates has been demonstrated using biomaterials such as DNA, proteins and viral particles, there are still significant challenges in achieving ordered and higher level architectures. Previously described methods are limited by the generation of randomly distributed nanoparticles with inconsistent diameters and inter-particle distances. The filament-connector assemblies are ideal templates for the synthesis and alignment of nanomaterials in complex arrangements. We therefore proposed to attach nanoparticles in a defined and ordered manner through the addition of a hexa-histidine sequence to the C-terminus of γPFD. Subsequently, we examined whether nickel-coated gold nanoparticles could be attached at high density to the γPFD to create 1D gold arrays (Fig. 4a). The refolded histidine-tagged filaments were incubated with an equal molar amount of 1.8- or 5-nm nickel-nitrilotriacetic acid (Ni^2+^-NTA)-gold nanoparticles for 8 h. The resulting structures were imaged by TEM, which revealed that the 1.8- and 5-nm gold nanoparticles were arranged into ordered 1D arrays along filaments (Fig. 4b). Filament cross-linking was observed in samples containing the 5-nm nanoparticles, presumably due to the multiple Ni-NTA on an individual 5-nm nanoparticle^22^ attaching numerable filaments (Fig. 4c). This cross-linking could be avoided by addition of a fivefold molar excess of 5-nm Ni^2+^-NTA-nanogold, followed by a gentle centrifugation to pellet and isolate the linear filament-nanoparticle arrays from the free nanogold (Supplementary Fig. 10). The dense alignment and spacing of gold nanoparticles along the filament was as expected for each subunit attached to a nanoparticle.

Moreover, the 1D metallic nanoparticle arrays on the filaments can be subsequently grown into continuous metallic nanowires. This was achieved by deposition of gold onto the existing nanoparticles using the catalytic effects of gold surfaces to selectively reduce gold from chloroauric acid in the presence of sodium borohydride (Fig. 4d). Growth of the 1D nanoparticle arrays into continuous nanowires was verified by TEM and scanning electron microscopy studies, which revealed a progressive growth of the nanowires with increasing deposition time. Continuous nanowires obtained after 2 min of deposition have an average diameter of 46 nm and a length equal to the underlying filament template.

Continuous nanowires with specific length uniformity could also be created by capping filaments with the TERM-(E-coil) at specific ratios before templating (Fig. 4e). To generate metallic and conductive nanoarchitectures, the TERM-(E-coil) capped filaments were assembled with a hexa-histidine TERM-(K-coil)-foldon three-way connector, coated with gold nanoparticles (Supplementary Fig. 10) and grown into continuous metallic pinwheels (Fig. 4f). These results demonstrate a versatile approach to control the assembly and overall dimensions of nanowire structures that could be used for the fabrication of nanodevices over a wide range of processing conditions.

To investigate the potential of these biotemplated nanowires as electronic components in future nanoelectronic devices, electrical transport studies were performed on individual nanowires. The electrical resistance of the continuous gold nanowires was measured after assembly across 2.5-μm gap gold electrodes patterned on silicon wafers (Fig. 4g) using AC dielectrophoresis (DEP)^23^. Two-terminal current–voltage (*I*–*V*) measurements of the nanowires showed a linear *I*–*V* relationship with an estimated resistivity of 6.45 × 10^−7^ Ωm (Fig. 4h). The nanowire resistance is greater than that of bulk gold (2.24 × 10^−8^ Ωm), but significantly more conductive than previous Au nanowires templated on actin^24^, Au-DNA nanowires^25^ or Au-CNT hybrid structures^26^. No conductivity was detected for filaments not assembled to gold nanoparticles. The breakdown behaviour of the nanowires was examined as a function of applied voltage. With an increase in bias voltage, the scan showed irreversible breakage of the conducting nanowire at 34 V (Fig. 4i), possibly due to the disconnection of the gold nanowire originated from electron scattering and joule heating.

## Discussion

In summary, we have used protein shape heuristics and protein–protein interface engineering to create novel protein templates for the positioning of nanoparticle arrays. This work demonstrates the possibility of molding proteins into new architectures so far not found in nature, and suggests a means to generate novel three-dimensional assemblages. Furthermore, these ultrastable biotemplates are ideal mesoscale structures on which to build functional materials with unique properties. The extreme stability exhibited by the connectors in conjunction with the γPFD expands the range of potential processing conditions that can be employed for the fabrication of nanodevices beyond the limits imposed by the labile nature of conventional biomolecules. In addition, the γPFD and connector proteins may be easily produced in microorganisms, suggesting a synchronous and complementary role with the emerging field of synthetic biology to rapidly build protein patterns of nanoscale dimensions for biomaterials. We envisage that the strategies developed in this work will be generally applicable for the design of more elaborate and robust protein shapes suitable for conjugation to form functional materials.

## Methods

### Protein design and molecular modelling

Protein models for γPFD and TERM as described previously^9^,^11^ were joined together or with foldon (PDB accession 2IBL) along the helical peptide backbones. The TERM-(E- and K-coil) models were created using the I-TASSER suite^27^ with the γPFD model as a template. Subsequently, the structures were refined by molecular dynamic simulations. All-atom molecular dynamic simulations were performed as described previously^11^ using the YASARA software package^28^. Briefly, the AMBER force field^29^ was used with a cutoff distance of 7.86 Å for van der Waals interactions and long-range electrostatic interactions calculated using the Particle Mesh Ewald algorithm^30^. The model had a periodic boundary cell created so that all atoms were at least 50 Å distance from walls and filled with explicit water to a density of 0.997 g l^−1^. Initial models were built with either the two- or three-way connectors attached to short four-subunit γPFD filaments. The model structures were energy-minimized, followed by a simulated annealing until energy improved by <0.05 kJ mol^−1^ per atom during 200 steps. Water molecules were then converted into sodium and chloride until a NaCl concentration of 0.9% was obtained. Initial protein models were equilibrated for 200 ps with temperature kept at 300 K by rescaling atom velocities using a Berendsen thermostat^31^. Subsequently, the models were simulated for an additional 2 ns at either 300 or 353 K, and the flexibility of the residues analysed by measuring the root mean square fluctuations (RMSF) of the residue C^α^ atoms.

### Protein expression and purification

The gene sequences for the connector proteins and FRET reporters were synthesized (DNA 2.0) and cloned into the bacterial expression vector pJexpress414 (DNA 2.0). Plasmid DNAs encoding wild-type γPFD, hexa-histidine gPFD and TERM have been described previously^8^,^11^. The plasmids were transformed into BL21 T7 Express *E. coli* (NEB) and grown at 37 °C in Luria Broth containing 100 μg ml^−1^ ampicillin to an optical density of 0.6 at A_600_. Subsequently, protein expression was induced for 15 h at 25 °C with the addition of 0.1 mM isopropylthiogalactoside. Cells were lysed and centrifuged at 22 000*g* for 40 min to clear the soluble protein. The γPFD and TERM proteins were purified as described previously^8^,^11^. The connector proteins containing a histidine-tag were purified by affinity chromatography on Ni-NTA resin (Invitrogen). Connector proteins containing a Strep-tag were purified by affinity chromatography by incubation on Streptactin sepharose (IBA GmbH). The mCerulean3 and mVenus fusion proteins were purified initially by hydrophobic interaction chromatography using a Toyopearl Butyl-650C resin column (TOSOH Bioscience). The mCerulean3 and mVenus fusion proteins were further purified by ion-exchange chromatography on a Q Sepharose fast flow column (GE Life sciences). The purity of eluted protein fractions were determined by visual inspection of an SDS–PAGE gel stained with SimplyBlue (Invitrogen). Purified proteins were dialyzed overnight against several changes of dialysis buffer (20 mM NaH_2_PO_4_ pH 7.5, 100 mM NaCl), concentrated using Amicon Ultra centrifugal filter columns (Millipore) and lyophilized for storage.

### Assembly of filaments and connectors

Lyophilized protein stocks were solubilized in 8 M guanidinium-HCl, 10 mM NaH_2_PO_4_, pH 8.0, and protein concentration determined by a Bradford assay. The connectors and filaments were assembled together by mixing the denatured proteins at varying molar ratios followed by a rapid dilution in PBS and incubation at 40 °C for 16 h. Subsequently, the refolded filament/connector assemblies were incubated with either Ni-NTA or Streptactin resin for 3 h and washed extensively with PBS to remove unbound proteins. The filament/connector assemblies were eluted from the Ni-NTA or Streptactin resin using 500 mM imidazole or 10 mM desthiobiotin, respectively. To demonstrate the assembly of filaments to the connectors, the eluted filament-connector assemblies were dialyzed against PBS to remove guanidinium-HCl, and run on a 10–20% gradient SDS-PAGE gel (Invitrogen) with Spectra BR protein ladder (Thermo Scientific). Native PAGE was used to examine the binding of TERM-(E-coil) to TERM-(K-coil) and the ability of these proteins to function as capping proteins for γPFD filaments. TERM-(E-coil) and TERM-(K-coil) were combined together in equimolar ratios or with γPFD, refolded and run on 4–12% gradient Tris-glycine PAGE gels (Invitrogen) with a NativeMark protein standard (Invitrogen). Filaments capped with the TERM-(E-coil) were separated into uniform sizes on a HiPrep 26/60 Sephacryl S-200 HR size-exclusion column (GE Life Sciences) as described previously^11^.

### Thermal stability of engineered connectors

The secondary structure and thermal stability of filaments and the engineered connector proteins were examined by far-UV circular dichroism. Protein samples were refolded at 9 μM for 4 h at 25 °C in 20 mM NaH_2_PO_4_ pH 8.0 with a final guanidinium-HCl concentration normalized to 260 mM for all samples. The concentrations of refolded proteins were verified using a Bradford assay. The circular dichroism spectra were obtained using a Jasco J-810 spectrometer (Jasco) by averaging three wavelength scans from 200 to 260 nm in 0.1 nm steps at 25 °C, with signal averaging time of 2 s and a bandwidth of 1 nm. Subsequently, the samples were heated from 25 °C to 100 °C at a rate of 1 °C min^−1^, and ellipticity measured at 208 and 222 nm in 1 °C intervals. The results are expressed as the mean residue molar ellipticity in degrees cm^2^ dmol^−1^. Equilibrium denaturation curves for the foldon domain of the three-way connector were measured by monitoring the intrinsic fluorescence emission of the sole tryptophan residue of foldon. The refolded three-way connector was dialyzed against PBS to remove all traces of guanidinium-HCl, diluted to a final concentration of 3 μM in PBS, and degassed for 1 h. Subsequently, the proteins were heated from 25 to 100 °C at 1 °C 2 min^−1^ in a quartz cuvette and intrinsic fluorescence emission between 310 and 400 nm collected using a 295 nm excitation wavelength on a Horiba Jobin Yvon Fluoromax-4 spectrofluorometer equipped with a thermally controlled cell holder. To examine the thermal stability of the three-way connector trimer, the refolded three-way was dialyzed against PBS to remove guanidinium-HCl, loading buffer (300 mM Tris-HCl pH 6.8, 6% SDS, 40% glycerol, 500 mM DTT, 0.02% bromophenol blue) added to a final SDS concentration of 1%, incubated at varying temperatures of 25–100 °C for 5 min, and run on a 10–20% gradient SDS–PAGE gel.

### Imaging of connector assemblies

Samples for TEM were prepared by diluting the filament/connector assemblies in PBS at 20 μg ml^−1^ and deposited onto 400-mesh carbon/formvar coated copper grids (Electron Microscopy Sciences) for 2 min at room temperature. The sample was wicked-off and grid washed with nanopure water, and stained with 2% uranyl acetate. Grids were visualized using either a Tecnai T12 120 kV TEM (FEI) or a Tecnai TF20 200 kV TEM (FEI). The digitized TEM images were analysed using the ImageJ public-domain software (US National Institutes of Health) to measure filament length, and Student's *t*-tests performed to determine statistical significance.

### FRET binding assays

A steady-state FRET binding assay was used to determine the equilibrium binding constants (*K*_*d*_) for the TERM-(E-coil) and TERM-(K-coil), and within the TERM dimer. The fluorescent fusion proteins TERM-(E-coil)-mCerulean3 and TERM-(K-coil)-mVenus were refolded in the presence of a 50-fold excess of TERM to block the available X1 binding site for 24 h at 25 °C in refolding buffer (50 mM Tris-HCl pH 7.5, 18 mM NaCl, 8 mM KCl, 400 mM L-arginine, 1 M guanidinium-HCl, 1 mM EDTA, 10% glycerol, 5 mM dithiothreitol). Serial dilutions of TERM-(K-coil)-mVenus were dispensed in duplicate into the wells of black 96-well plates with final concentrations ranging from 8–0 μM. An equal volume of 2 μM TERM-(E-coil)-mCerulean3, mCerulean3 or buffer was dispensed into the wells, mixed and incubated for 2 h. Fluorescence was measured in a SpectraMax plate reader (Molecular Devices) using a 400 nm excitation, 420 nm cutoff filter, and emission scan of 450–600 nm. At the 530 emission peak intensity, dilution and direct excitation controls were subtracted from the TERM-(E-coil)-mCerulean3/TERM-(K-coil)-mVenus titrations and the binding curves analysed as described previously^20^. Measurements were repeated for the TERM-mCerulean and TERM-mVenus dimerization using the same methodology.

### Nanowire assembly and conductivity measurements

Gold nanoparticle labelling of the three-way connector was performed by maleimide-labelling of cysteine residues. A three-way connector containing a C-terminal cysteine residue was refolded in a reducing buffer of 20 mM NaH_2_PO_4_ pH 6.5, 150 mM NaCl, 2 mM EDTA, 5 mM DTT and buffer exchanged using NAP-5 columns (GE Life Sciences) into 20 mM NaH_2_PO_4_ pH 6.5, 150 mM NaCl, 2 mM EDTA. Mono-maleimido nanogold particles (Nanoprobes) were suspended in 200 μl of water and added to the connector for 3 h at 25 °C. Subsequently, γPFD filaments were added to the connector/nanogold mixture, incubated for 24 h and bound to Ni-NTA affinity resin. The resin was washed three times with PBS to remove unbound gold nanoparticles, and the connector-filament assemblies eluted using PBS containing 500 mM imidazole. The eluted assemblies were added to TEM grids, stained with HQ silver enhancement (Nanoprobes) to increase the contrast of the gold particles, rinsed thoroughly with deionized water, and stained with 1% uranyl acetate.

Metal nanoparticles were arrayed onto filament and connector assemblies by refolding the proteins to a final concentration of 1.2 μM, and adding 1.2–12 μM of nanoparticles in the form of either 1.8- or 5-nm Nickel-NTA-Nanogold (Nanoprobes). The gold-templated filaments were separated from the free nanoparticles by centrifugation at 500 *g*, suspended in PBS, and applied to Formvar/Carbon grids. To grow the nanoparticle arrays on the filaments into continuous metallic nanowires, the grids containing the nanoparticle-coated filaments were incubated in 0.2 mM HAuCl_4_+0.5 mM NaBH_4_, washed with water and air dried. Gold-arrayed Ni-NTA-nanogold nanowires were carefully dropped on the 2.5 μm electrode in a 100% humidity chamber to prevent water evaporation. An AC frequency of 10 MHz was applied using an HP 31320A waveform function generator at 10 V peak-to-peak amplitude. The DEP was terminated when the in-loop voltameter read decreased in resistance. This ensures the DEP forms a single nanowire channel for the resistivity measurement. The DEP assembled samples were copiously washed with deionized water and isopropyl alcohol and dried in a vacuum at 30 °C for 30 min. Resistance and breakdown behaviour were measured on a Keithley semiconductor characterization system model 4200 using a two-wire resistance measurement function. Scanning electron microscopy images of nanowires assembled across electrodes were obtained on an FEI Quanta eSEM operating at 10 kV and at a working distance of 10 mm.

### Data availability

The data that support the findings of this study are available from the corresponding author on request.

## Additional information

**How to cite this article:** Glover, D. J. *et al*. Geometrical assembly of ultrastable protein templates for nanomaterials. *Nat. Commun.* 7:11771 doi: 10.1038/ncomms11771 (2016).

## Supplementary Material

Supplementary Information Supplementary Figures 1-10
