# Supplementary material for: Geometrical assembly of ultrastable protein templates for nanomaterials
Source: Nat Commun. 2016 Jun 1;7:11771. doi: 10.1038/ncomms11771 (PMC4895442; doi:10.1038/ncomms11771)
Supplement: Supplementary Information — Supplementary Figures 1-10 [file ncomms11771-s1.pdf]

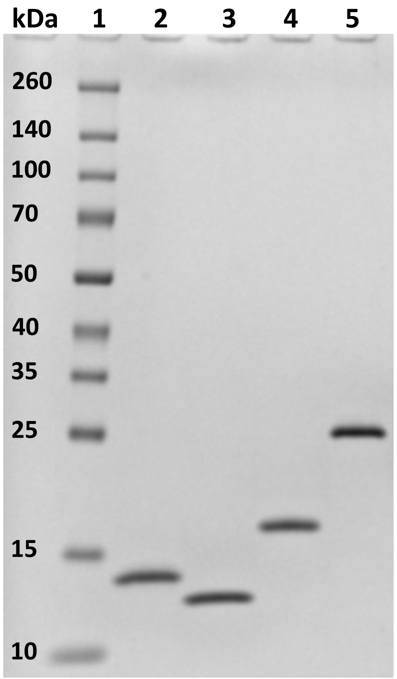

Lane:

1. Spectra BR protein ladder
2. γPFD
3. TERM
4. 3-way connector
5. 2-way connector

Supplementary Figure 1. **SDS-PAGE of bacterially expressed and purified proteins.** A representative gel of proteins purified via the four different techniques used in this study. The γPFD protein purified by ion-exchange chromatography followed by hydrophobic interaction chromatography, the hexa-histidine-tagged TERM protein purified using nickel-NTA affinity chromatography, and the strep-tagged 2-way and 3-way connector proteins purified by Streptactin affinity chromatography.

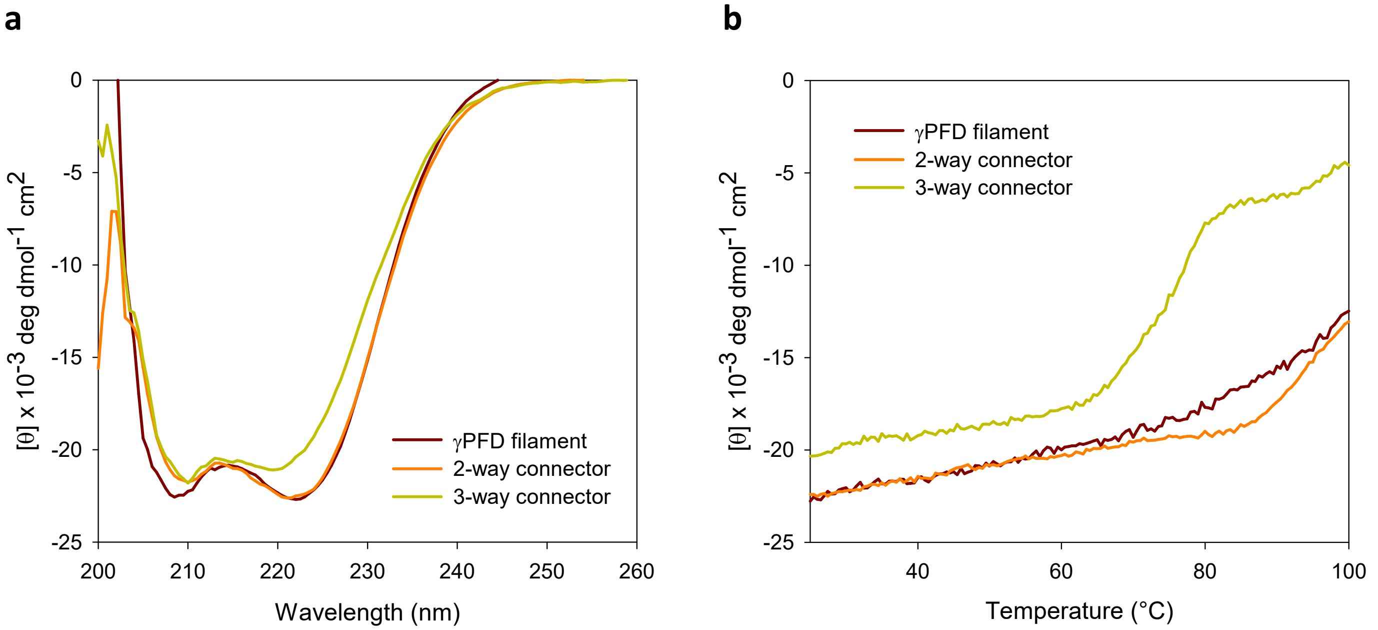

Supplementary Figure 2. Secondary structure and thermal stability of the γPFD and connector proteins. a, Far-UV circular dichroism spectra of γPFD and the 2-way and 3-way connectors. All the proteins had a predominantly helical conformation with minima near 208 and 222 nm. **b**, The thermal stability of the γPFD and connector proteins was measured by thermal ramps performed by heating from 25°C to 100°C at a rate of 1°C/min. Ellipticity was measured at 222 nm in 1°C intervals.

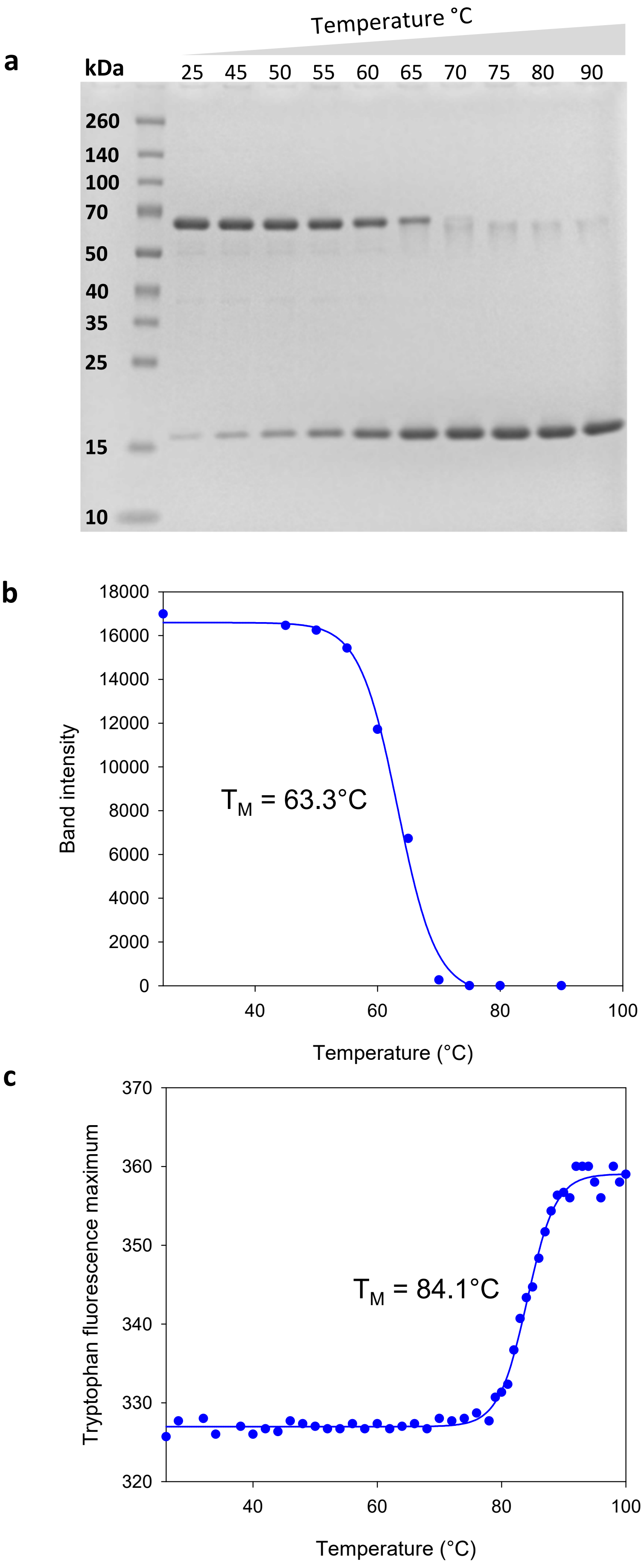
a
Temperature °C
kDa
25 45 50 55 60 65 70 75 80 90
260
140
100
70
50
40
35
25
15
10
b
Band intensity
T_M = 63.3°C
Temperature (°C)
c
Tryptophan fluorescence maximum
T_M = 84.1°C
Temperature (°C)

Supplementary Figure 3. The 3-way connector trimerizers into highly thermal-stable assemblies. **a**, SDS-PAGE gel analysis of the 3-way connector demonstrates a trimeric structure that is denatured by high temperature into monomers. The samples in 2% SDS were incubated at varying temperatures for 5 min. **b**, The intensity of the trimer bands in **a** were quantified by densitometric analysis. The data were fitted to a sigmoidal curve and had an apparent T_m of 63.3 ± 0.5°C. **c**, Unfolding curve of the foldon domain within the 3-way connector was recorded by measuring the fluorescence emission maxima of the sole tryptophan residue of foldon at an excitation wavelength of 295 nm as a function of increasing temperature. The data are the means of three separate experiments and had an apparent T_m of 84.1 ± 0.2°C when fitted to a sigmoidal curve.

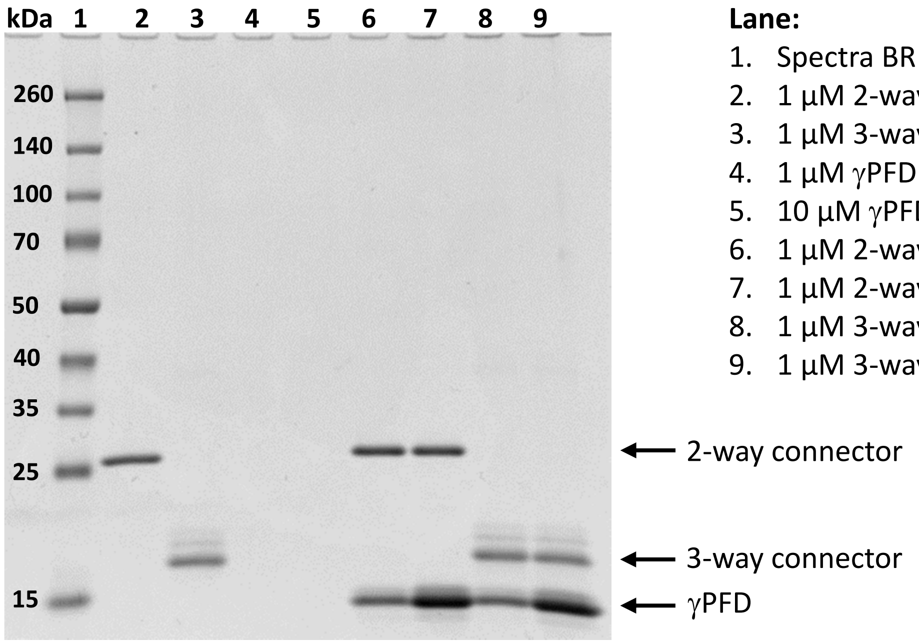

Lane:

1. Spectra BR protein ladder
2. 1 μM 2-way connector
3. 1 μM 3-way connector
4. 1 μM γPFD
5. 10 μM γPFD
6. 1 μM 2-way connector + 1 μM γPFD
7. 1 μM 2-way connector + 10 μM γPFD
8. 1 μM 3-way connector + 1 μM γPFD
9. 1 μM 3-way connector + 10 μM γPFD

Supplementary Figure 4. The engineered connector proteins attach to γPFD filaments. An SDS-PAGE gel was used to show the binding of the two- and three-way connector proteins to γPFD filaments following a pull-down assay. The two-way and three-way connectors contain a strep-tag and bind Streptactin resin; however, an equal or 10-fold molar excess of γPFD does not bind the resin and is washed away. When the connector proteins are refolded in the presence of an equal or 10-fold molar excess of γPFD, the connectors attach to both γPFD and the Streptactin, resulting in co-elution of both proteins.

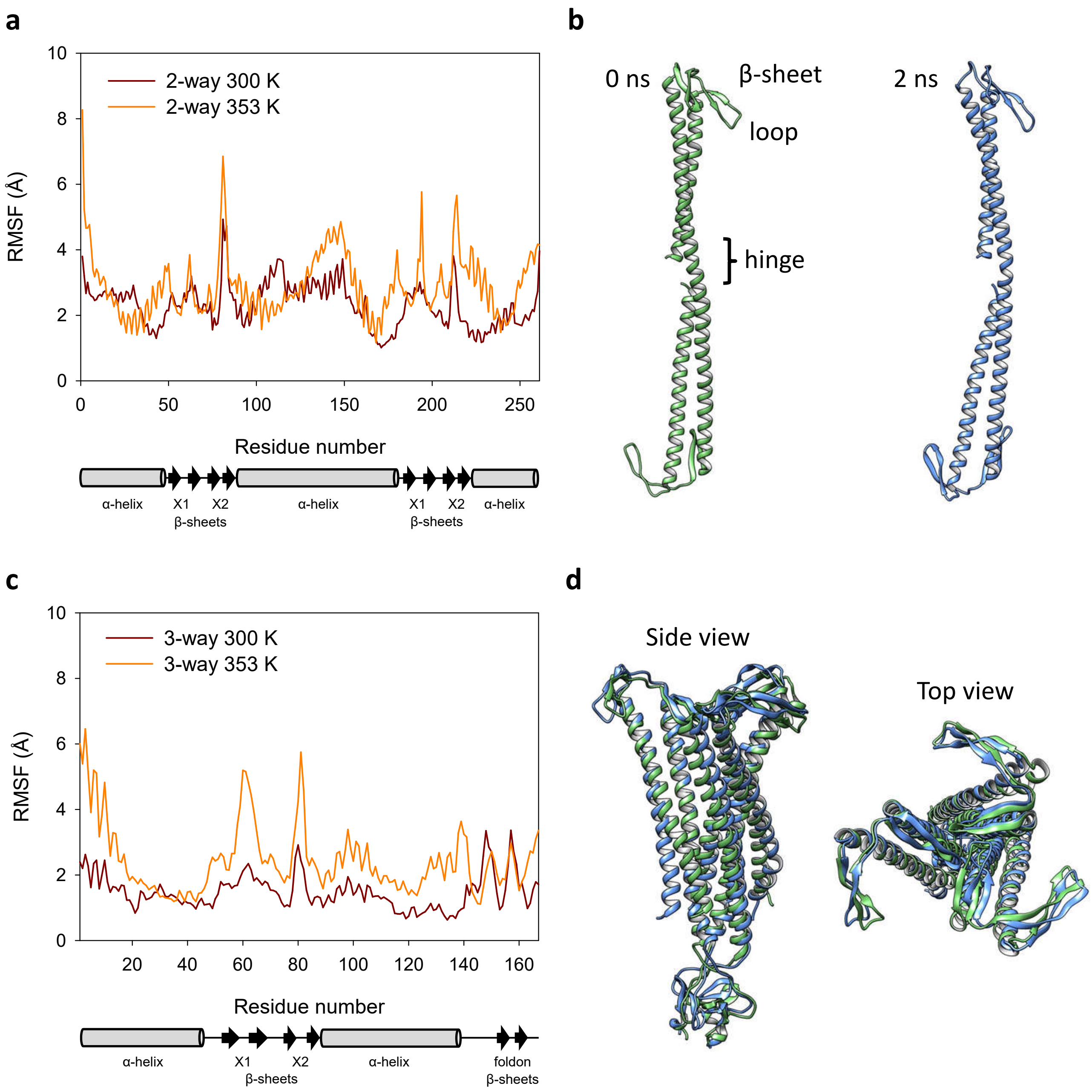

Supplementary Figure 5. The structural flexibility of the engineered connector proteins during all-atom molecular dynamic (MD) simulations. All simulations were performed at 300K or 353K for 2 ns. **a**, The root mean square fluctuations (RMSF) of C^{α} atoms in the two-way connector during the simulation. **b**, Protein models of the two-way connector at the start of the simulation (green) and after 2 ns simulation (blue). **c**, The RMSF of the three-way connector during the simulation. **d**, Superimposed structure of the initial three-way connector model (green) and after 2 ns simulation (blue).

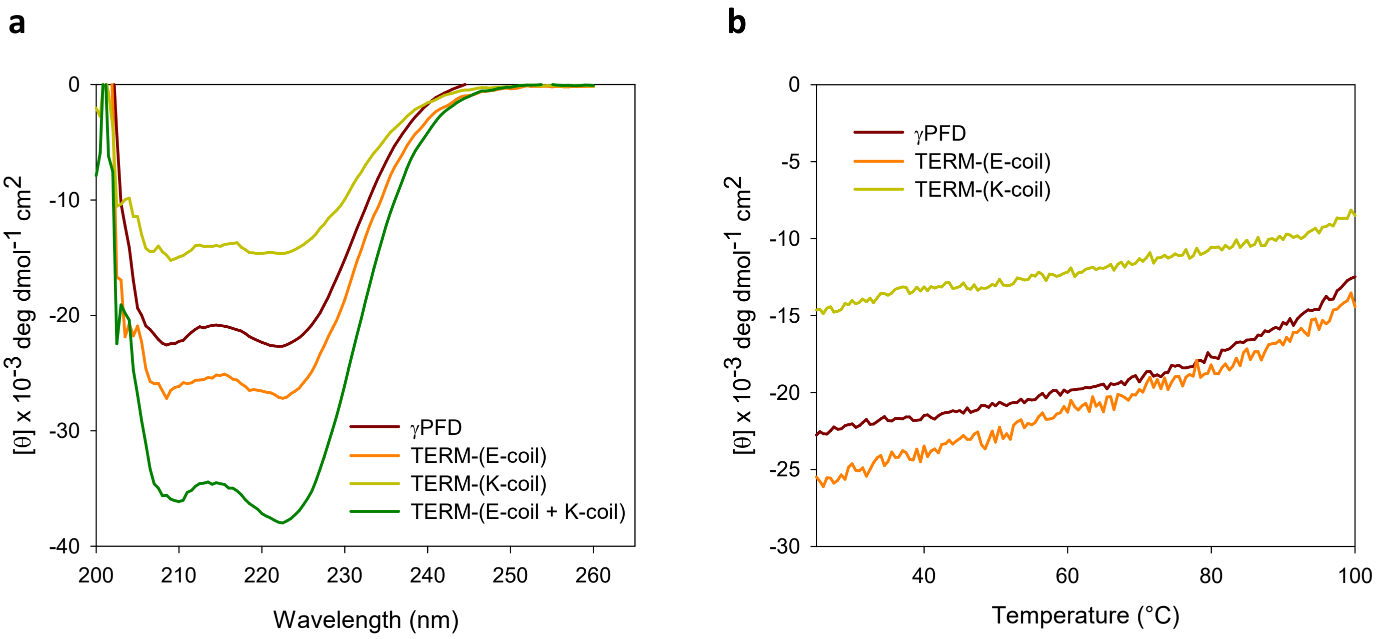

Supplementary Figure 6. The secondary structure and thermal stability of TERM-(E-coil) and TERM-(K-coil). a, Far-UV circular dichroism spectra of γPFD, and the TERM-(E-coil) and TERM-(K-coil) proteins either individually or combined. **b**, The thermal stability of γPFD, TERM-(E-coil), and the TERM-(K-coil) was measured by a thermal ramp performed by heating from 25°C to 100°C at a rate of 1°C/min. Ellipticity was measured at 222 nm in 1°C intervals.

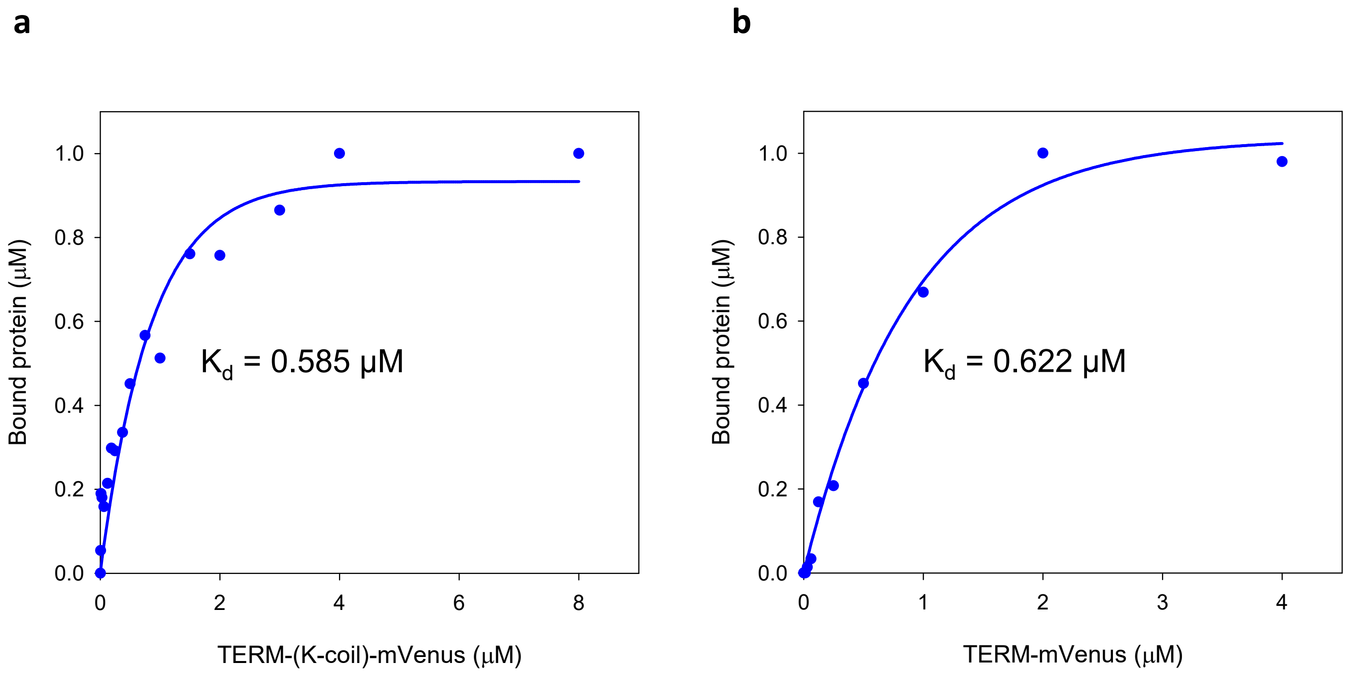

Supplementary Figure 7. Determination of equilibrium binding constant (*K_d*). a, The K_d between TERM-(E-coil)-mCerulean3 and TERM-(K-coil)-mVenus. Bound protein determined from FRET data as a function of free TERM-(K-coil)-mVenus concentration, fitted by a hyperbola with B_{max} = 1 μM TERM-(E-coil)-mCerulean3 and K_d = 0.585 μM. **b**, The K_d between the TERM dimer (TERM-mCerulean3 and TERM-mVenus) using steady-state FRET assays. Bound protein determined from FRET data as a function of free TERM-mVenus concentration, fitted by a hyperbola with B_{max} = 1 μM TERM-mCerulean3 and K_d = 0.622 μM. Data for all measurements are the means of three separate experiments.

a

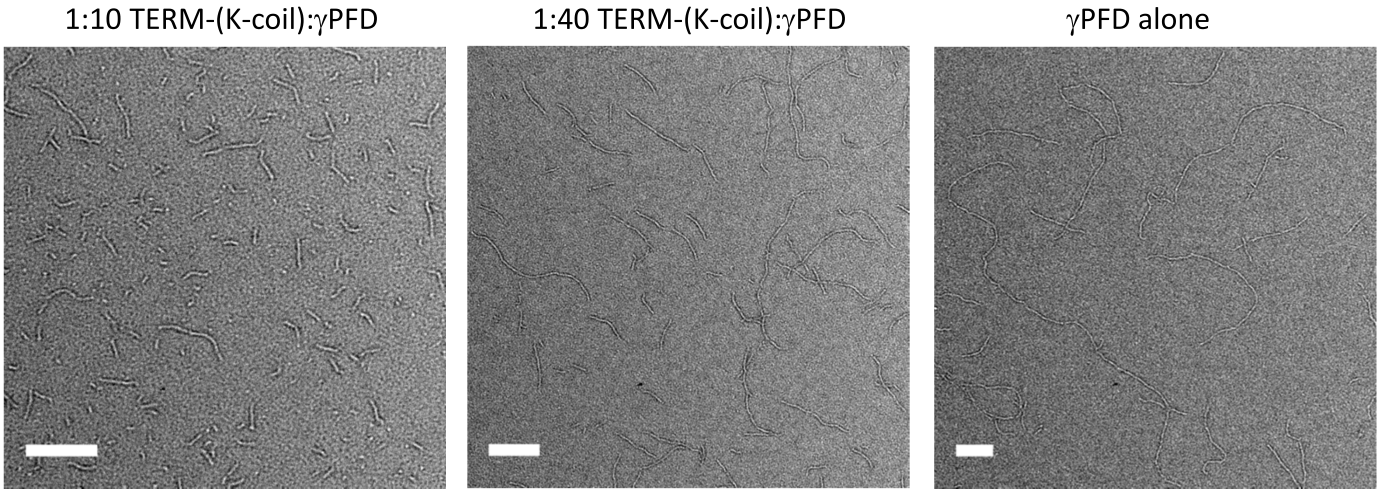

b

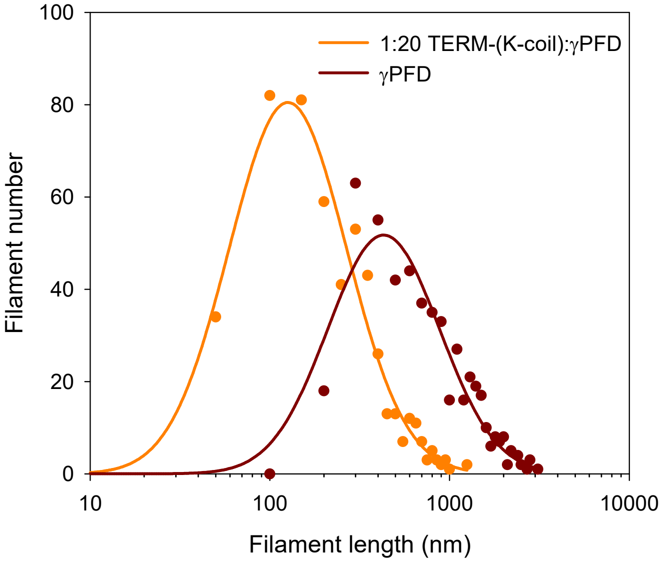

Supplementary Figure 8. The length of γPFD filaments can be controlled by the engineered bait-prey proteins. a, Denatured γPFD was mixed with various ratios of the TERM-(K-coil) protein, refolded and imaged by TEM. Scale bars = 250 nm. **b**, Filament lengths were measured in digitized TEM images such as those in **a** and plotted as distributions of length (*n*=500 filaments).

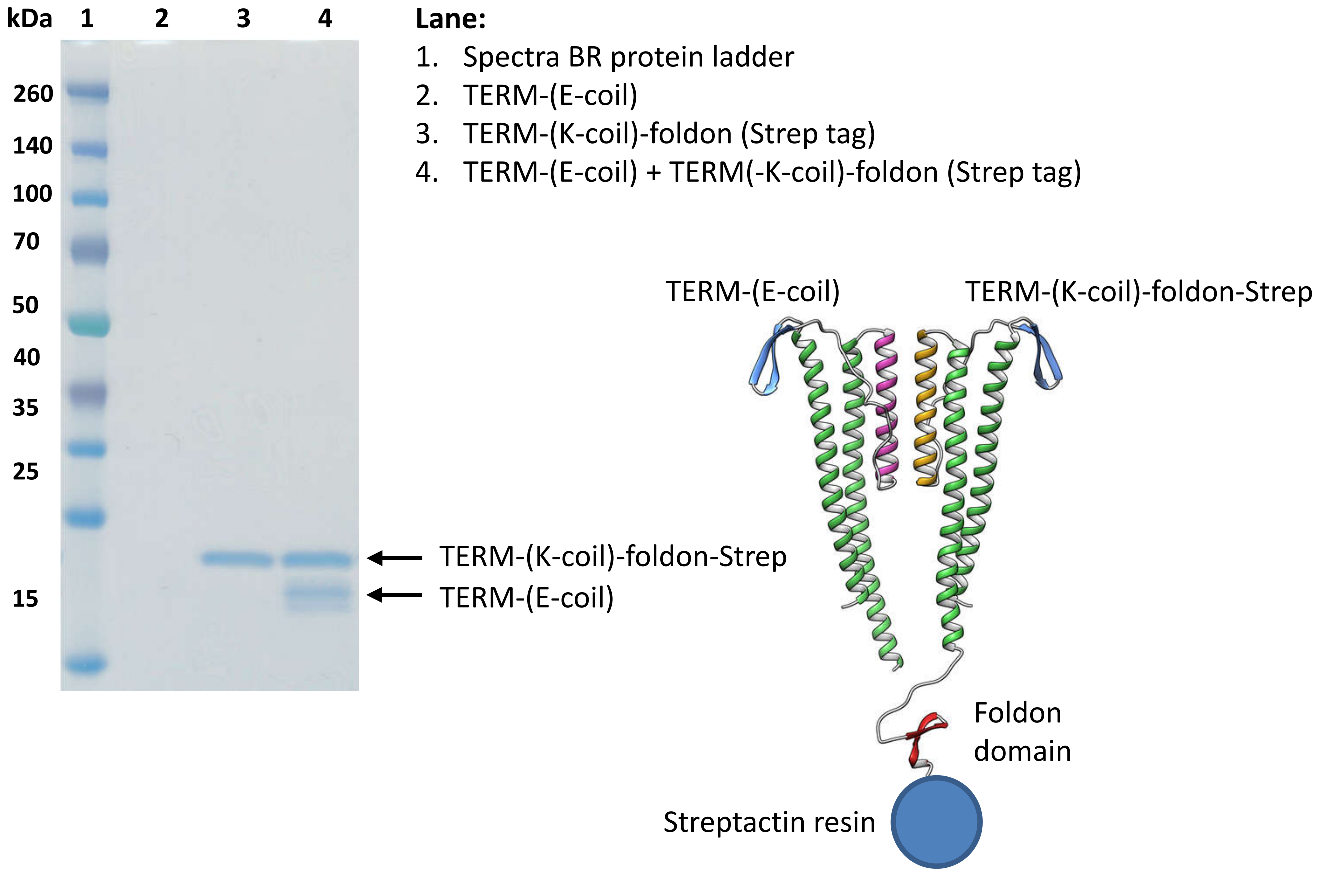

Supplementary Figure 9. The engineered TERM-(K-coil) binds to a three-way connector that contains the TERM-(E-coil). A pull-down assay was used to show that the TERM-(K-coil) protein was able to bind to the three-way connector containing the TERM-(E-coil) sequence and a strep-tag affinity sequence. The TERM-(E-coil)-foldon three-way connector was able to bind to Streptactin resin, thereby pulling along the bound TERM-(K-coil) which lacks the strep tag. Subsequently, the eluted proteins were examined on an SDS-PAGE gel.

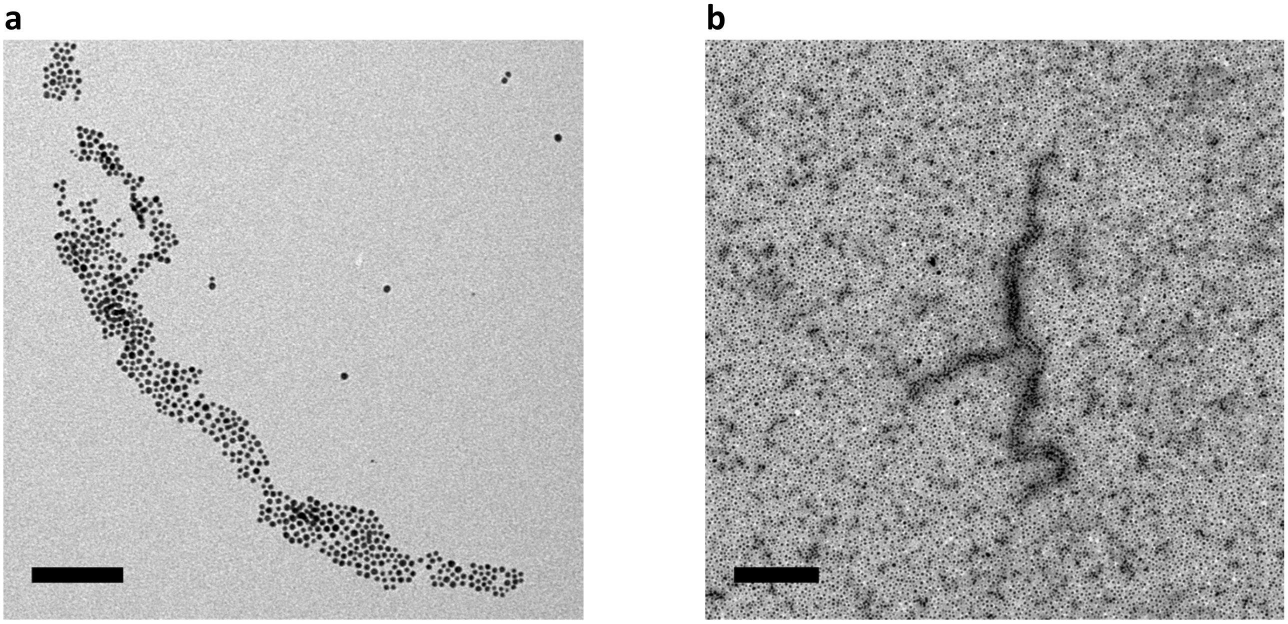

Supplementary Figure 10. Nanoparticle arrays on protein filament templates. a, TEM of 5-nm gold nanoparticles assembled along a γPFD filament. Incubation of a 5-fold molar excess of gold nanoparticles prevented cross-linking between individual filaments (scale bar = 100 nm). **b**, The three-way connector was used to assemble three filaments that could subsequently be templated with gold nanoparticles into defined structures (scale bar = 200 nm).
